# The effect of artificial saliva on the control of dry mouth: a semi-experimental study on COVID-19 patients under Non-invasive mechanical ventilation

**DOI:** 10.1186/s12903-024-04386-4

**Published:** 2024-05-31

**Authors:** Ladan Sedighi, Sorour Khari, Morteza Hasheminik, Atefe Salimi Akinabadi, Haleh Alipour, Elnaz Shafigh, Navid Shafigh

**Affiliations:** 1 Department of Medical and Surgical Nursing, School of Nursing and Midwifery, Clinical Research Development Center, Shahid Modarres Educational hospital, Shahid Beheshti University of Medical Sciences, Tehran, Iran; 2 Master of Critical Care Nursing, School of Nursing and Midwifery, Shahid Beheshti University of Medical Sciences, Tehran, Iran; 3 Department of Nursing, Sabzevar Branch, Islamic Azad University, Sabzevar, Iran; 4 Master of Medical Surgical Nursing, Clinical Research Development Center, Shahid Modarres Educational Hospital, Shahid Beheshti University of Medical Sciences, Tehran, Iran; 5 Department of Pediatric Pulmonology, Mofid Children’s Hospital, Shahid Beheshti University of Medical Sciences, Tehran, Iran; 6 Operative Dentistry Department, Faculty of Dentistry, AJA University of Medical Sciences, Tehran, Iran; 7 Critical Care Quality Improvement Research Center, Shahid Modarres Hospital, Shahid Beheshti University of Medical Sciences, Tehran, Iran

**Keywords:** COVID-19, Non-invasive mechanical ventilation, Artificial saliva, ICU patients

## Abstract

**Objectives:**

Since maintaining oral hygiene is essential in nursing care, the present study was conducted to determine the effect of oral care using Mucosamin artificial saliva spray to control dry mouth in ICU patients with COVID-19.

**Materials and methods:**

The current semi-experimental research was conducted on eighty patients with COVID-19 selected using the available sampling method. The study tool was a Beck oral assessment scale (BOAS). The case and control groups were selected from two hospitals with relatively similar conditions and treatment procedures. For patients in the intervention group, mucosamin artificial saliva spray was used in addition to the common care, while control group patients received only common care.

**Results:**

Eighty patients were randomly assigned to two groups named control and intervention (40 patients in each group). The intervention was very effective in reducing the BOAS score after four days in comparison with the control group (9.23 vs. 12.05, respectively; p-value < 0.001). Based on the adjusted model, the application of artificial saliva reduced the BOAS score, indicating improvement in mouth dryness. While the BOAS score was increased in the control group, it had a declining trend in the intervention one.

**Conclusion:**

The study’s results showed that using artificial saliva spray could effectively reduce the symptoms of dry mouth in patients with COVID-19 treated with non-invasive mechanical ventilation.

**Clinical relevance:**

The present study introduced an applicable solution (artificial saliva) to treat mouth dryness in ICU patients under mechanical ventilation.

## Introduction

A prevalent impaired oral health is reported among COVID-19 patients [1]. This is related to several mechanisms such as cytokine storm or hypercytokinemia, an immune response more related to viral infections and most often remerging after viral reparatory syndromes’ pandemic [2, 3]. Besides, saliva cell glands are highly affected by the coronavirus, especially in those patients with oxygen therapy. It is also believed that better oral health (improved salivary condition) is related to best treatment achievement in COVID-19 patients [1, 4, 5].

Dry mouth is a disorder caused by insufficient secretion of saliva, changes in saliva quality, and the dysfunction of the salivary glands and dental cavity [6–8]. During the COVID-19 epidemic, dry mouth was recognized as one of the complications caused by this disease [9]. In patients with COVID-19, dry mouth occurs due to changes in the quantity and composition of oral saliva. This disease is associated with oral manifestations caused by direct viral infection, simultaneous infections, drug reactions, and stress; other symptoms may include oral soft tissue ulcers, gingivitis, plaques, erythema, and changes in salivary glands and oral nerves [10]. On the other hand, putting patients with COVID-19 under non-invasive mechanical ventilation increases the symptoms of dry mouth and ulcers in these patients [11].

Non-invasive positive pressure ventilation (NIPPV) helps improve patients’ breathing and oxygenation by providing a combination of air and oxygen using positive pressure and a mask. The use of non-invasive mechanical ventilation has increased significantly in the last two decades and has decreased cases of invasive mechanical ventilation and mortality [12]. With the start of the COVID-19 pandemic, non-invasive mechanical ventilation has become more prevalent in all intensive care units around the world [11], and its use in the treatment of patients is associated with a high degree of success. However, on the other hand, its use is associated with side effects [13]. Mouth dryness is one of the main complaints of patients under non-invasive mechanical ventilation, and the patients report it as an unpleasant and annoying feeling [14, 15].

Due to the importance of oral care, studies have been conducted to compare and evaluate the performance of different types of mouthwashes and different types of oral moisturizers [15]. Besides, most of the interventions are applied to assess the effect of tooth brushing or oral care procedures in ICU patients. The implications of these interventions are very difficult, especially for ICU patients with severe conditions and patients with worse oral health [16, 17]. However, So far, no study has been conducted to evaluate the effectiveness of artificial saliva as an easily applied procedure in improving oral complications caused by non-invasive mechanical ventilation in patients with COVID-19. As a possible solution for dry mouth, artificial saliva, with the trade name (VA-oralube), containing sorbitol and carboxymethyl cellulose, could be evaluated for this end. Artificial saliva has a neutral pH and, due to the presence of fluoride, causes remineralization of the surface of the teeth without any side effects. Artificial saliva has the same biological and physical properties as saliva and moisturizes the dry tissue of the mouth [18]. Therefore, the present study aims to determine the effect of oral care using Mucosamin artificial saliva spray to control dry mouth in patients with COVID-19 who are under non-invasive mechanical ventilation.

## Materials and methods

This semi-experimental study was conducted on the patients with COVID-19 under non-invasive mechanical ventilation in two hospitals for six months from April to September 2022. The clinical trial registration code is IRCT20220223054107N1. The trial was firstly registered on 25/02/2022.

Eligible patients who met the inclusion criteria were selected from the intensive care unit of two hospitals. Patients of one of the hospitals were assigned to the control group and the patients of another hospital were considered as the intervention group. In the intervention group, in addition to the usual care, Mucosamin artificial saliva spray was used, and in the control group only usual care was provided.

Demographic information and the results of the initial evaluation of the patient’s mouth were recorded. Each patient was followed up for three days and evaluated daily. Patient mouth examinations were performed at four stages: before starting non-invasive mechanical ventilation, day one: one day after, and day three: 3 days after. The patient was taught to keep the artificial saliva spray in his mouth and then swallow it, maintaining moisture in the oral cavity and throat.

The main purpose of the study was to determine the effect of Mucosamin artificial saliva spray on mouth dryness. According to the results of previous studies [9], the sample size was determined with a type 1 error rate of 0.05, a power of 80%, and 10% attrition.

Inclusion criteria were conscious patients hospitalized in the intensive care unit with COVID-19 (having a positive CT scan or PCR test) under non-invasive mechanical ventilation who have not used non-invasive mechanical ventilation before, aged between 18 and 70, and GCS above 13. Also, patients who died during the research or whose oxygen therapy method was changed, patients who were taking drugs related to decreased salivary function (including anticholinergic agents, antihistamines, antihypertensive drugs, antiparkinson drugs, antipsychotics and antidepressants, diuretics, and muscle relaxants), patients with diseases causing dehydration such as diarrhea, patients with mental illness, patients with noticeable oral lesions, plague, patients with autoimmune diseases, patients with dentures, pregnant ones, and patients with a history of smoking were excluded from the study.

### Data collection tools

#### Personal profile questionnaire (PPS)

The PPS was created by the researcher with the validity confirmed by ten nursing professors regarding age, gender, educational status, marital status, underlying disease, history of smoking, medications, case number, and hospitalization date of patients, the number of hours’ non-invasive mechanical ventilation was used per day which the researcher then completed.

#### Beck oral assessment scale (BOAS scale)

This scale was used to assess the patient’s oral condition. This scale provides a realistic and clinical assessment of the mouth of critically ill patients [19]. The validity and reliability of the tool have been confirmed by the study of Safarabadi and Rezaei. This tool consists of 5 sub-groups, which include the examination of (1) Lips, (2) Gums and oral mucosa, (3) Tongue, (4) Teeth, and (5) Saliva. Each is graded into four parts and scored from 1 to 4. The scoring range is from 5 (no oral dysfunction) to 20 (severe oral dysfunction), and a score of more than five is considered abnormal. The lower the score indicates better oral health (absence of problems and disorders), the higher the score indicates impaired oral health. In this way, a score of 5 means no disorder, a score of 6–10 means mild disorder, a score of 11–15 means moderate disorder, and a score of 16–20 means severe disorder.

### Study procedure

The participants of this study were laboratory-confirmed SARS-CoV-2 patients admitted in two hospitals in Tehran. One hospital was designated as the intervention group, while the other served as the control group. This two hospital approach was utilized to minimize bias in the results. Both hospitals were referral ones working under the supervision of Shahid Beheshti University of Medical Sciences; they accepted COVID-19 patients in any stage of disease and had the same oral care protocols, all patients who met the inclusion criteria were selected from the intensive care unit of two hospitals. Patients of a hospital assigned to the control group and the patients of another hospital were considered as the intervention group. In the intervention group, in addition to the usual care, Mucosamin artificial saliva spray was also used, and in the control group, only the usual care was given. Routine oral care for both hospitals included using 0.2% chlorhexidine mouthwash and toothbrushes.

The situation of the nurse car in 2 hospital were the same because these 2 hospital are educational hospitals and same protocol carried out in them. Sampling was carried out by two trained ICU nurses, who started collecting the samples at the same time. In order to avoid the influence of intervening variables and to standardize the routine oral care in the two hospitals, the frequency, time, and method of mouthwash and oral nursing care in both hospitals were examined by the researcher to ensure the standardization of the oral care method. During a training program, all nurses were taught how to wash mouths and perform oral care, and they were asked to perform oral care based on the training and written protocol provided to them. The protocol taught to the nurses regarding the observance of routine oral hygiene in the test and control groups was such that before washing the mouth with chlorhexidine, the nurse would use a soft toothbrush to brush the entire surface of the mouth, gums, tongue, pharynx, and the internal and external surfaces of the teeth, gums, then use chlorhexidine mouthwash. This protocol was implemented every 12 h in the same way for patients under non-invasive mechanical ventilation in intervention and control groups. Conductors of the research appeared at the bedside of patients wearing personal care equipment and monitored the performance of nurses in each hospital during the study.

In both hospitals, on the first day of patients’ use of non-invasive mechanical ventilation, demographic information (age, gender, underlying disease, medications, and smoking history) and the results of the initial evaluation of the patient’s mouths were recorded. Each patient was recorded for three days and examined daily. Examining and evaluating patients’ mouths was performed during four stages (day zero: before the start of non-invasive mechanical ventilation, first day: one day after using non-invasive mechanical ventilation, and third days after using non-invasive mechanical ventilation) with the help of study tools. Moreover, the patient was taught to hold artificial saliva in his mouth and then swallow it, thus maintaining moisture in the oral cavity and throat [17].

### Statistical analysis

Descriptive statistics were presented using mean ± standard deviation (SD) for numeric variables and frequency (percentage) for categorical variables by group (control and intervention). Fisher exact tests were used to test the relationship between group and demographic variables. Generalized estimation equation (GEE) was used to examine the impact of each demographic and clinical variable on the BOAS score over time. The GEE was also used to evaluate the effect of intervention on the BOAS score over time, controlling for confounders. The mean of BOAS on each day was compared in both groups using the independent t-test. Furthermore, the GEE was used to assess the change in BOAS over time by group. The line and trajectory plots were used to illustrate the average and patient-specific changes in BOAS score over time by group (control and intervention), respectively. Line plots were also used to indicate the proportion and its 95% confidence interval for each level of categorized BOAS by time and group. The 95% confidence interval (CI) for proportions was calculated using Newcombe methods [20]. Analyses were conducted using R (version 4.2.1) and SPSS (version 26, SPSS Inc., Chicago, IL, United States). Statistical significance was defined as a p-value less than 0.05.

## Results

### Patients’ demographic and clinical characteristics

**Fig. 1:**
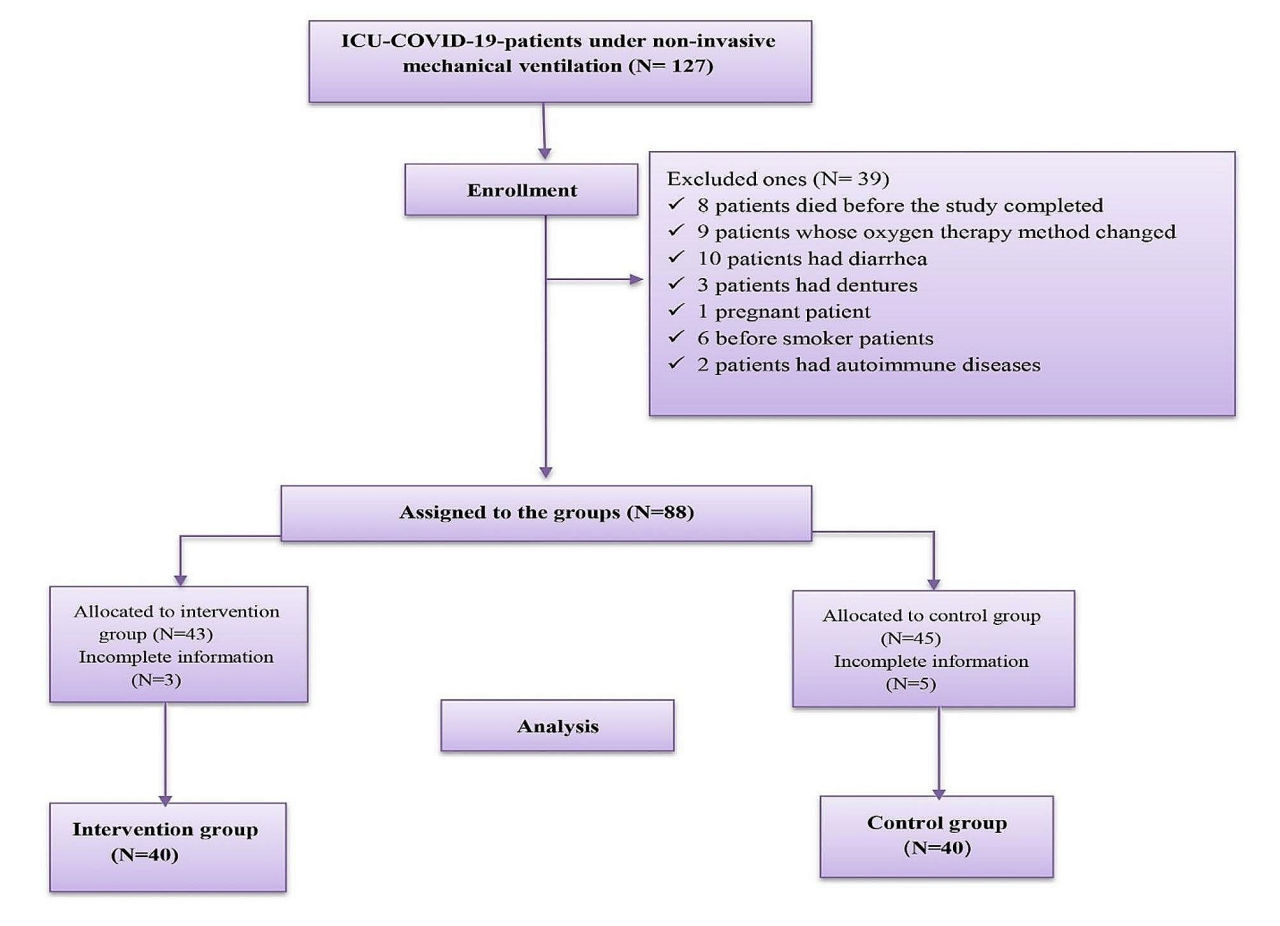
Consort diagram

From 127 COVID-19 ICU patients enrolled in the study, 88 patients were assigned to control and intervention groups. Then, the information of 80 patients (40 patients in each group) was collected for the final analysis (Fig. 1).

The patients with a mean age (± SD) of 66.37 ± 13.97 years were included in the study. Forty-three participants were female (53.75%), and 37 were male. The most frequent comorbidities were hypertension (45.00%), diabetes mellitus (32.50%), lung disease (23.75%), and cardiovascular disease (21.25%). The overall mean duration of non-invasive ventilation was 15.15 ± 4.50 h. No significant difference was observed in the groups (control and intervention) in relation to demographic and clinical variables (Table 1).

**Table 1:** Demographic and clinical characteristics of the patient population in the dataset

Variables	Total (*N* = 80)	Group		*P*-value
		Intervention (*N* = 40)	Control (*N* = 40)	
Age, Mean ± SD	66.33 ± 13.97	67.65 ± 13.28	65.00 ± 14.67	0.400
Sex, N (%)				0.654
Male	37 (46.25%)	20 (50.00%)	17 (42.50%)	
Female	43 (53.75%)	20 (50.00%)	23 (57.50%)	
Diseases, N (%)				1.000
Yes	66 (82.50%)	33 (82.50%)	33 (82.50%)	
No	14 (17.50%)	7 (17.50%)	7 (17.50%)	
Diabetes mellitus, N (%)				0.812
Yes	26 (32.50%)	12 (30.00%)	14 (35.00%)	
No	54 (67.50%)	28 (70.00%)	26 (65.00%)	
Hypertension, N (%)				0.500
Yes	36 (45.00%)	20 (50.00%)	16 (40.00%)	
No	44 (55.00%)	20 (50.00%)	24 (60.00%)	
Cardiovascular disease, N (%)				0.274
Yes	17 (21.25%)	6 (15.00%)	11 (27.50%)	
No	63 (78.75%)	34 (85.00%)	29 (72.50%)	
Lung disease, N (%)				1.000
Yes	19 (23.75%)	10 (25.00%)	9 (22.50%)	
No	61 (76.25%)	30 (75.00%)	31 (77.50%)	
Malignancy, N (%)				1.000
Yes	2 (2.50%)	1 (2.50%)	1 (2.50%)	
No	78 (97.50%)	39 (97.50%)	39 (97.50%)	
Kidney disease, N (%)				1.000
Yes	13 (16.25%)	6 (15.00%)	7 (17.50%)	
No	67 (83.75%)	34 (85.00%)	33 (82.50%)	
Thyroid disease, N (%)				0.359
Yes	5 (6.25%)	1 (2.50%)	4 (10.00%)	
No	75 (93.75%)	39 (97.50%)	36 (90.00%)	
Neurological disease, N (%)				0.518
Yes	11 (13.75%)	7 (17.50%)	4 (10.00%)	
No	69 (86.25%)	33 (82.50%)	36 (90.00%)	
Drug history, N (%)				0.439
Yes	60 (75.00%)	28 (70.00%)	32 (80.00%)	
No	20 (25.00%)	12 (30.00%)	8 (20.00%)	
Duration of non-invasive ventilation, Mean ± SD	15.15 ± 4.50	14.93 ± 4.62	15.38 ± 4.42	0.657

### Relation between Beck oral assessment scale score and demographic or clinical variables

The effect of each demographic and clinical variable on the BOAS score over time (before intervention, first day after intervention, second day after intervention, and third day after intervention) was presented in Table 2. Accordingly, significant effects of malignancy (β 1.62; 95% CI 0.39, 2.85), thyroid disease (β −0.26; 95% CI −0.47, −0.04), and the use of non-invasive mechanical ventilation (β 0.06; 95% CI 0.02, 0.09) on the BOAS score over time were declared. Furthermore, as the use of non-invasive mechanical ventilation increased over time, BOAS also rose (Table 2).

**Table 2:** The impact of demographic and clinical data on the BOAS over time

Parameter	Β (95% CI)	*P*-value
Age	0.01 (−0.02, 0.03)	0.519
Time	1.44 (0.68, 2.19)	< 0.001
Age * Time	0.00 (−0.01, 0.01)	0.944
Sex: Male vs. Female	−0.23 (−0.94, 0.48)	0.526
Time	1.43 (1.20, 1.66)	< 0.001
[Male vs. Female] * Time	0.07 (−0.27, 0.41)	0.688
Disease: Yes vs. No	0.55 (−0.32, 1.42)	0.217
Time	1.24 (0.90, 1.58)	< 0.001
[Disease: Yes vs. No] * Time	0.28 (−0.10, 0.67)	0.152
Diabetes mellitus: Yes vs. No	0.60 (−0.24, 1.43)	0.162
Time	1.41 (1.23, 1.60)	< 0.001
[Diabetes mellitus: Yes vs. No] * Time	0.21 (−0.17, 0.60)	0.284
Hypertension: Yes vs. No	−0.07 (−0.80, 0.67)	0.855
Time	1.49 (1.27, 1.71)	< 0.001
[Hypertension: Yes vs. No] * Time	−0.05 (−0.39, 0.29)	0.754
Cardiovascular disease: Yes vs. No	−0.32 (−1.28, 0.65)	0.517
Time	1.43 (1.24, 1.62)	< 0.001
[Cardiovascular disease: Yes vs. No] * Time	0.18 (−0.25, 0.60)	0.411
Lung disease: Yes vs. No	0.56 (−0.39, 1.51)	0.251
Time	1.45 (1.27, 1.62)	< 0.001
[Lung disease: Yes vs. No] * Time	0.08 (−0.39, 0.55)	0.735
Malignancy: Yes vs. No	0.45 (−2.97, 3.87)	0.796
Time	1.48 (1.32, 1.64)	< 0.001
[Malignancy: Yes vs. No] * Time	1.62 (0.39, 2.85)	**0.010**
kidney disease: Yes vs. No	−0.09 (−1.03, 0.85)	0.853
Time	1.47 (1.28, 1.66)	< 0.001
[kidney disease: Yes vs. No] * Time	−0.06 (−0.43, 0.32)	0.768
Thyroid disease: Yes vs. No	0.41 (−0.57, 1.39)	0.413
Time	1.47 (1.29, 1.65)	< 0.001
[Thyroid disease: Yes vs. No] * Time	−0.26 (−0.47, −0.04)	**0.022**
Neurological disease: Yes vs. No	0.15 (−1.04, 1.34)	0.804
Time	1.46 (1.28, 1.64)	< 0.001
[Neurological disease: Yes vs. No] * Time	0.03 (−0.49, 0.54)	0.919
Drug history: Yes vs. No	0.69 (−0.05, 1.43)	0.067
Time	1.42 (1.11, 1.72)	< 0.001
[Drug history: Yes vs. No] * Time	0.06 (−0.30, 0.43)	0.741
The number of hours of non-invasive mechanical ventilation	0.04 (−0.03, 0.12)	0.255
Time	0.65 (0.08, 1.21)	0.025
The number of hours of non-invasive mechanical ventilation * Time	0.06 (0.02, 0.09)	**0.002**

### Comparing the Beck oral assessment scale score between groups over time

The mean of the BOAS score over time in both groups was summarized in Table 3. It was found that the BOAS score of the intervention group was significantly lower than that of the control group before intervention (intervention vs. control: 5.70 vs. 6.60; p-value = 0.001). In contrast, the average BOAS in the intervention group was significantly higher than that of the control group on the first day following the intervention (12.50 vs. 9.40; p-value < 0.001). On the second day following the intervention, BOAS scores were equal in the groups (10.70 vs. 10.93; p-value = 0.313). However, the BOAS score was lower for the intervention group than that of the control group on the last day (9.23 vs. 12.05; p-value < 0.001).

**Table 3:** Comparing the mean BOAS score between control and intervention

Variable	Total	Group		*P*-value
		Control	Intervention	
BOAS score, before intervention	6.15 ± 1.35	6.60 ± 1.60	5.70 ± 0.85	0.001
BOAS score, the first day after intervention	10.95 ± 2.60	9.40 ± 1.92	12.50 ± 2.25	< 0.001
BOAS score, second day after intervention	10.81 ± 2.04	10.93 ± 2.02	10.70 ± 2.09	0.313
BOAS score, third day after intervention	10.65 ± 2.50	12.05 ± 2.21	9.25 ± 1.93	< 0.001
P-value (GEE)	< 0.001	< 0.001	< 0.001	

The impact of the intervention on the changes in BOAS score over time was adjusted for hours of non-invasive mechanical ventilation, malignancy, and thyroid disease (variables with a significant relationship with the Beck score). As presented in Table 4, the reduction in the BOAS score was more quickly over time for the intervention group than for the control group, adjusting for the confounders (β −2.98; 95% CI −3.37, −2.59). This change in BOAS score over time was more clearly illustrated; the average score was lower in the intervention group before intervention and three days after intervention. In addition, the trajectory plot displays how the BOAS score changes over time for each patient (Fig. 1).

**Table 4:** The impact of the intervention on the BOAS over time controlling the no. of hours of non-invasive ventilation, malignancy, and thyroid disease

Parameter	Β (95% CI)	*P*-value
No. of the hours of non-invasive mechanical ventilation	0.08 (0.01, 0.15)	0.031
Malignancy: Yes vs. No	1.39 (−2.39, 5.18)	0.471
Thyroid disease: Yes vs. No	0.19 (−0.30, 0.68)	0.448
Using artificial saliva: Yes vs. No	2.80 (1.98, 3.62)	< 0.001
Time	1.41 (1.14, 1.68)	< 0.001
[Using artificial saliva (Intervention): Yes vs. No] * Time	−2.98 (−3.37, −2.59)	< 0.001

* time on the BOAS, controlling for other significant variables

### Comparing the Beck oral assessment scale proportion between groups over time

**Fig. 2:**
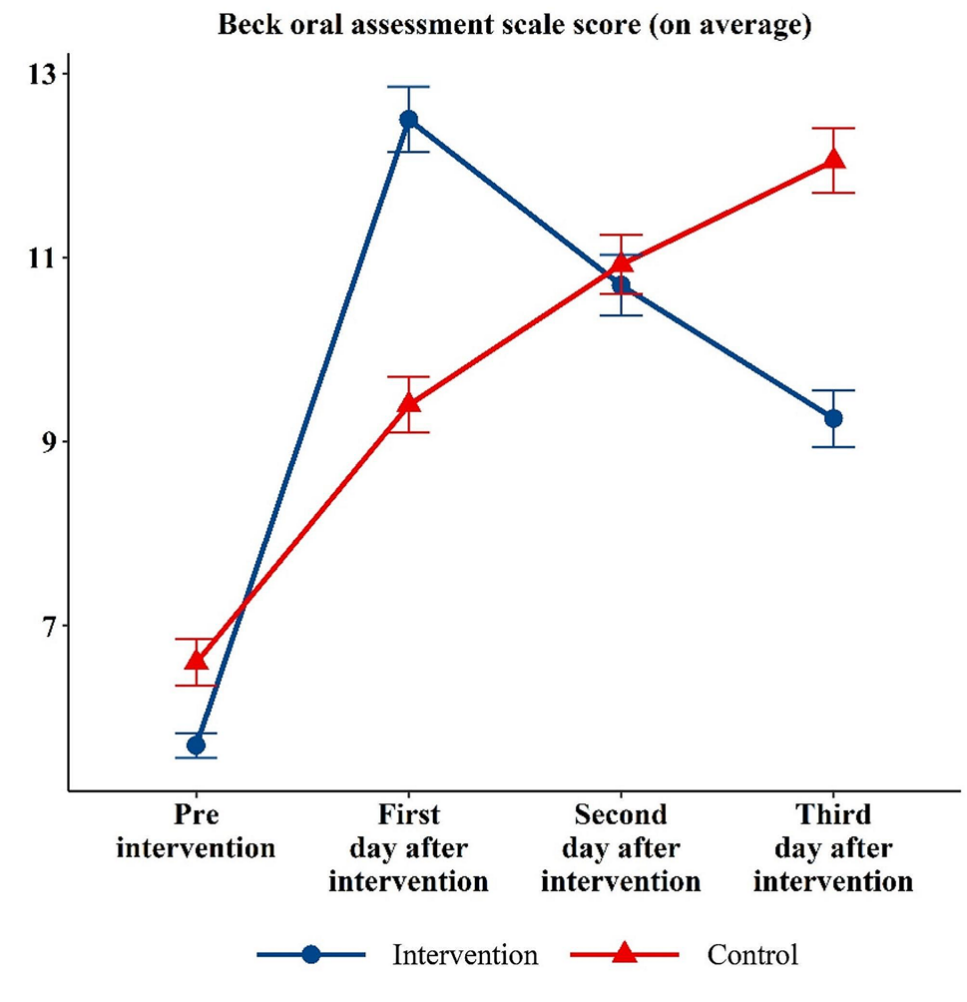
Changes in BOAS scores over time for each group

Figure 2 shows the change in the average BOAS scores in the control and intervention groups during the three days of study. Over time, the BOAS score of the control group sharply increased; however, the average score of the patients in the intervention group increased in the first day and the score remarkably decreased in the second and third days.

**Fig. 3:**
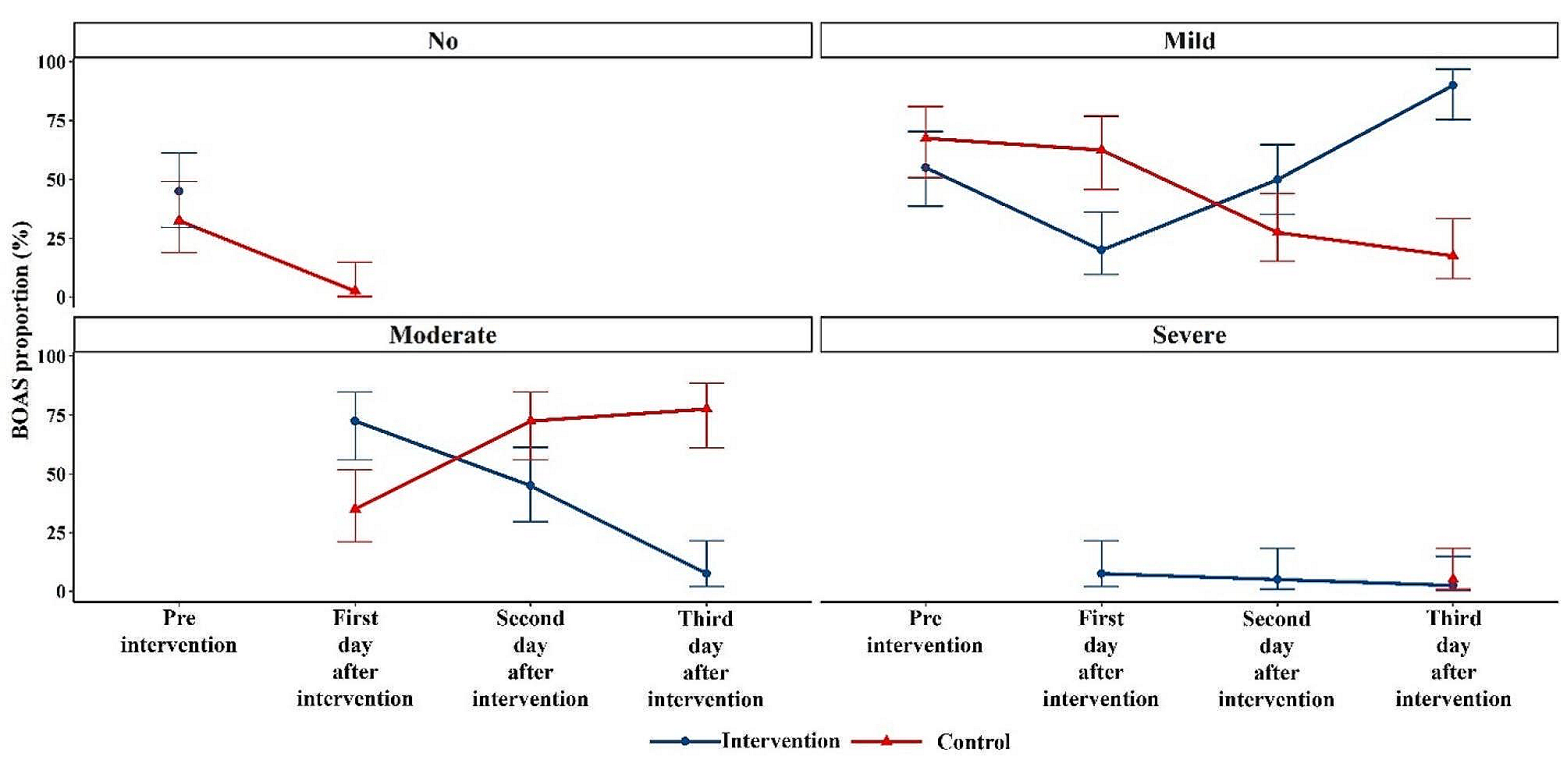
The proportion of categorized BOAS by time and group

Also, the BOAS score is reported based on the severity of the patients in Fig. 3. Accordingly, the proportion of mild BOAS increased in the intervention group and decreased in the control group. By contrast, the proportion of moderate BOAS increased in the control group and decreased in the intervention group over time.

## Discussion

This study aimed to determine the effect of oral care using artificial saliva on the control of dry mouth in patients with COVID-19 under non-invasive mechanical ventilation. The results represented that the intervention (using Mucosamin artificial saliva spray) improved oral health in the COVID-19 UN-IMW patients. In other words, the mouth dryness in the patients of the intervention group was lower than that of the control group. It can indicate the effectiveness of artificial saliva spray in reducing dry mouth. Studies also confirm the effectiveness of artificial saliva in improving oral health and the quality of life-related to oral health in patients [21]. In the study by Nuchit et al. (2020) conducted on patients with head and neck cancer after radiotherapy, artificial saliva was associated with a reduction in dry mouth [22]. In fact, in patients with head and neck cancer under radiation therapy, the most effective intervention to reduce dry mouth is artificial saliva [14, 22]. The use of artificial saliva compared to a placebo in patients with diabetes was also associated with a reduction in dry mouth [23]. Unlike other viral and bacterial respiratory illnesses, COVID-19 patients typically do not have excessive airway secretions. These studies are consistent with the present study regarding the effect of artificial saliva spray in reducing dry mouth. The present study was conducted on patients with COVID-19 under non-invasive mechanical ventilation, which was challenging [24].

In patients with COVID-19, viral disease, secondary inflammatory response to viral infection, opportunistic infections, stress, and lack of oral hygiene are the most common factors that cause dry mouth [11]. In Baiadsee et al.’s study, 72% of patients with COVID-19 complained of dry mouth [25]. In addition, infection with COVID-19 is also associated with changes in saliva secretion and dry mouth, among which patients with COVID-19 admitted to the hospital and undergoing non-invasive and invasive mechanical ventilation are exposed to a severe decrease in saliva and dry mouth [10], these findings align with the present study since the patients in the test and control groups had dry mouths before the intervention.

Control of dry mouth in patients with cancer and undergoing radiotherapy is done through frequent drinking of water and using drugs that stimulate saliva production [22]. Meanwhile, in patients with COVID-19 under non-invasive mechanical ventilation due to poor respiratory status, the possibility of repeatedly removing the mask to reduce the sensation of dry mouth is the main challenge in using this type of oxygen therapy [11]. Therefore, for patients in the test group, Mucosamin spray was used to prevent dry mouth in addition to the usual oral care. After the intervention, the BOAS scale score decreased significantly compared to the control group, indicating better oral and dental health conditions in these patients. In the study by Pico-Orozco et al., many patients treated with CPAP mentioned dry mouth as a side effect that can negatively affect compliance with the treatment. Furthermore, it causes dryness of the oral mucosa. Traditionally, to prevent dry mouth, drinking sips of water in patients undergoing non-invasive mechanical ventilation has been suggested, which is against the goal of non-invasive mechanical ventilation treatment in patients [26]. These patients drink more water if they feel dry mouth, even though water does not have antimicrobial properties and does not maintain hydration and moisten the mouth for a long time. Therefore, using artificial saliva is a better solution, and this product is prepared with a viscosity higher than water and similar to the viscosity of natural saliva. It preserves oral tissues, facilitates speaking and eating, and reduces mucous membrane inflammation [14].

Despite the many innovations in producing comfortable masks for non-invasive mechanical ventilation, most patients under non-invasive mechanical ventilation still complain of dry mouth, which is associated with non-adherence to treatment [27]. Mouth breathing is another factor that influences the irritation and dryness of the mouth’s soft tissues. Based on the findings of the study by N Ingle et al., dry mouth caused by mouth breathing, oxygen therapy, and air being blown with pressure to the face can be improved by using an oral mucosa moisturizer and proper hydration of the oral tissue with the help of artificial saliva [22] which is in line with the findings of the study.

In the intensive care unit care program, brushing and using chlorhexidine in critically ill patients effectively prevents ventilator-associated pneumonia. However, it does not show evidence of dry mouth reduction [11]. This proves that in the present study, the patients of the control group who used the usual oral hygiene care, including chlorhexidine mouthwash and brushing teeth, had a poorer oral health status, and the ratio of average dry mouth increased in the control group. It decreased in the intervention group, which indicates the effectiveness of Mucosamin artificial saliva spray in reducing dry mouth. The results of Kvalheim’s study, conducted on patients admitted to the intensive care unit with dry mouth in the final stages of life, showed that patients prefer using 17% glycerol solution instead of artificial saliva because this product is cheaper and it is more accessible. On the other hand, patients mentioned that artificial saliva is more effective than glycerol solution [28].

Therefore, choosing the best treatment to relieve dry mouth symptoms will increase patients’ quality of life. Artificial saliva spray moistens the mouth’s surface, prevents excessive growth of pathogenic microorganisms, maintains the hardness of the oral structure, and has a long-term shelf life; it also softens the oral tissue, increases the mucous membrane’s moisture, accelerates wound healing, Slows down and reduces dry mouth [22, 29–31].

Based on the results of the present study, the duration of using non-invasive mechanical ventilation was one of the influential factors in causing dry mouth, which means that with the increase in the number of hours of using non-invasive mechanical ventilation, the amount of dry mouth in the test and control group patients increased. In Celik et al.’s study, continuous oxygen therapy through a mask and nasal cannula harmed oral health [32]. Considering this variable as a confounding variable and controlling it with statistical tests, it was observed that artificial saliva effectively reduces dry mouth in patients undergoing non-invasive mechanical ventilation by reducing the BOAS scale score in the test group patients.

According to the study’s results, the underlying disease of cancer is one of the factors that cause dry mouth. In Nasrollahi et al.’s study, mucositis and dry mouth had a significant relationship with radiotherapy [30]. In the present study, the patients were not undergoing chemotherapy and radiotherapy and were in the controlled stage of cancer. Considering this variable as a confounding variable and controlling it with statistical tests, it was observed that artificial saliva effectively reduces dry mouth in patients undergoing non-invasive mechanical ventilation by reducing the BOAS scale score in the test group patients. Also, in Nasrollahi et al.’s study, using Mucosamin three times a day for 72 h in patients with mucositis caused by radiotherapy was effective in improving mucositis, ulcers, and dry mouth compared to the control group [30].

In the current study, most patients were elderly, and complaining of dry mouth symptoms becomes an increasing problem with age [33]. Old age, non-invasive mechanical ventilation, and COVID-19 will increase this effect, causing dry mouth. In the study of Piaton et al., the use of artificial saliva three times a day before meals effectively reduced dry mouth and oral mucosa disorders in the elderly [21], which is in line with the present study.

In the present study, over time, the proportion of patients with mild dry mouth increased in the intervention group and decreased in the control group. The study by N Ingle et al. also showed that artificial saliva is clinically effective in treating dry mouth with immediate relief of symptoms [22]. Saliva is crucial for the health and proper functioning of the oral cavity. Despite many treatment options, such as stimulation or protection of the salivary glands, local artificial saliva replacement seems to be the most effective solution for reducing dry mouth [14].

### Limitations

We acknowledged that the number of included patients and the intervention group were limited due to financial and patient access restrictions. We could not assess the effect of other possible methods for the improvement of oral care in ICU patients with COVID-19. During the COVID-19 pandemic, access to hospitalized patients in the intensive care unit with severe pulmonary involvement who underwent noninvasive ventilation was very limited and there was a high risk of infection for the researchers, so only a few patients involved in the study.

## Conclusion

Taking into account the occurrence of dry mouth in many patients with COVID-19, in addition to treating the disease, attention should be paid to oral hygiene and control of dry mouth in these patients, even after their recovery. The study showed that artificial saliva spray could effectively reduce dry mouth symptoms in patients with COVID-19 treated with non-invasive mechanical ventilation. Considering that in the care of patients undergoing NIPPV, it is essential to use methods that improve oral health and prevent dry mouth with minimal complications and risk, it is necessary to increase the attention of health service providers regarding the need for oral care And nurses can use Mucosamin artificial saliva spray for this purpose when planning a care plan according to the needs of patients.

## Data Availability

The data that support the findings of this study are available from Shahid Beheshti University of Medical Sciences. Restrictions apply to the availability of these data, which were used under license for this study. Data are available from Dr. Navid Shafigh with the permission of Shahid Beheshti University of Medical Sciences.
